# Survival and Histopathological Study of Animals Bearing Ehrlich Tumor Treated With a Rhodium(II) Amidate

**DOI:** 10.1155/MBD.1999.17

**Published:** 1999

**Authors:** Breno Pannia Espósito, Szulim Ber Zyngier, Renato Najjar, Roberto Pinto Paes, Suely M. Ykko Ueda, José Carlos A. Barros

**Affiliations:** 1 University of São Paulo Institute of Chemistry Av. Prof. Lineu Prestes, 748 - r. 1265 São Paulo 055508-900 Brazil; 2 University of São Paulo Institute of Biomedical Sciences I Av. Prof. Lineu Prestes, 1524 São Paulo 055508-900 Brazil; 3 Faculty of Medical Sciences of the Santa Casa de São Paulo R. Da. Veridiana, 311 São Paulo 01238-010 Brazil

## Abstract

The survival of 90% of a tumor-bearing population treated with the complex Rh_2_ (CF_3_CONH)_4_ was examined and the pharmacological parameter *Surv*_90_ determined. Histopathological alterations raised for this drug in several tissues were studied in Balb-c mice. A *Surv*_90_ dose of 3.8x 10^-5^ mol/kg was found.


### SURVIVAL AND HISTOPATHOLOGICAL STUDY OF ANIMALS BEARING EHRLICH TUMOR TREATED WITH A RHODIUM(II) AMIDATE

Breno Pannia Espósito*[1a], Szulim Ber Zyngier*[1b], Renato Najjar[1a], Roberto Pinto Paes[2], Suely M. Ykko Ueda[2] and José Carlos A. Barros[2]

[1] University of São Paulo, São Paulo - 055508-900, Brazil
[a] Institute of Chemistry, Av. Prof. Lineu Prestes, 748 - r. 1265 <bpesposi@quim.iq.usp.br>
[b] Institute of Biomedical Sciences I, Av. Prof. Lineu Prestes, 1524
[2] Faculty of Medical Sciences of the Santa Casa de São Paulo,
R. Da. Veridiana, 311, São Paulo - 01238-010, Brazil

### ABSTRACT

The survival of 90% of a tumor-bearing population treated with the complex $Rh_2(CF_3CONH)_4$ was examined and the pharmacological parameter $Surv_{90}$ determined. Histopathological alterations raised for this drug in several tissues were studied in Balb-c mice. A $Surv_{90}$ dose of $3.8x10^{-5}$ mol/kg was found.

### INTRODUCTION

The use of rhodium (II) dimers as possible antitumoral agents has been investigated, and the recent literature reports a number of examples of these complexes which could overcome the toxicities of the carboxylates initially proposed [1]. Relatively few data are available about the biodistribution, pharmacokinetics and histopathology of the rhodium (II) dimer complexes. Souza and coworkers [2] investigated the distribution of rhodium in mice submitted to treatment with the adduct of rhodium propionate and sodium isonicotinate, by means of ICP-AES. Craciunescu and coworkers [3] performed a study of the biological activity, nephro, hepato and hematotoxicity of adducts of rhodium(II) and iridium(II) dimers with classical organic antimalarial drugs. Also, these authors described the renal histopathology after the administration of the complex $Rh_2(CH_3COO)_4(mepacrine)_2$.

Recently, the pharmacological parameters $IC_{50}$ (against Ehrlich ascites and U937 and K562 human leukemia cells) and $LD_{50}$ (in male Balb-c mice) of the complex $Rh_2(CF_3CONH)_4$ (tfacam) were reported. The $LD_{50}$ of tfacam was close to the value obtained for cisplatin in similar conditions (cf. [4] and references therein]. These results encouraged subsequent studies in the biological destinations of this and other rhodium (II) complexes. In this work, the pharmacological parameter $Surv_{90}$ was determined (defined as the dose that allows the survival of 90% of the tumor-treated animals [5]) of the drug tfacam. The histopathological study of brain, blood, kidney, spleen, liver, lungs, bone marrow, testes and ovary tissues from Balb-c mice treated with this complex are also presented.

### MATERIALS AND METHODS

The complex tfacam was synthesized and suspended in an aqueous solution (5% of Tween™-80) as described in [4].

a) Survival test: Male Balb-c mice were inoculated *i.p.* with $5x10^5$ cells of Ehrlich tumor and treated *i.p.* after 24 h with different volumes of a $1.2x10^{-3}$ M tfacam solution. Alive and dead animals were counted after 39 days.

b) Histopathological test: Fourteen healthy Balb-c mice were divided in three groups. Two animals received *i.p.* 500 μL of saline (control group). Seven animals received 500 μL of the aqueous Tween™-80 solution. Five animals received 500 μL of a $2.0x10^{-3}$ M tfacam solution so that each one of them had approximately the $Surv_{90}$ dose.

The mice were sacrificed to collect tissue (heart, lungs, blood, liver, kidneys, testes, ovary, brain and bone marrow) after 25 days. Those parts were kept in a 10% formol solution and then in blocks of paraffin and finally in slides to be observed in hematoxylin-eosin coloration in an optic microscope. No animals died for toxic effects of the drug during this period.

### RESULTS

a) Survival test: Table I shows the counts after the experimental period.

Table I: Determination of the parameter $Surv_{90}$

| Dose ($x10^{-5}$mol/kg) | Initial number | Dead after 39 days | % of survival |
|---|---|---|---|
| Control[a] | 7 | 7 | 0 |
| 1.0 | 7 | 2 | 71 |
| 1.5 | 8 | 2 | 75 |
| 2.1 | 5 | 1 | 80 |
| 2.6 | 8 | 1 | 84 |

[a] only Tween™--80

From these data, it was found a $Surv_{90}$ value of $3.8x10^{-5}$ mol/kg for the complex tfacam.

b) Histopathological test: No differences were observed between the control group and the group of animals that received only Tween™-80 solution.

The mice that received the rhodium drug showed the abnormalities reported in Table II.

Table II: Histopathological alterations observed in the mice treated with the $Surv_{90}$ dose of tfacam

| Animal | Histopathological alterations |
|---|---|
| 1 | no alterations |
| 2 | hepatic necrosis |
| 3 | hepatic necrosis; hemorragic points in the lungs; tubular necrosis in the kidneys |
| 4 | hepatic necrosis; pneumonia; tubular necrosis in the kidneys; focal brain dismielinization |
| 5 | tubular necrosis in the kidneys |

### DISCUSSION

Craciunescu *et al.* [3] used half of the $LD_{50}$ of the drug $Rh_2(CH_3COO)_4(mepacrine)_2$ and found alterations described as light to moderate nephrotoxicity, and no hepatotoxicity.

With the *i.p.* injection of tfacam solution in the range of 1.0 to $2.6x10^{-5}$ mol/kg, a linear dependence between the survival rate and the dose was obtained. The extrapolation to 90% afforded us the value of $Surv_{90} = 3.8x10^{-5}$ mol/kg. However, this dose is close to the $LD_{50}$ of the drug ($4.8x10^{-5}$ mol/kg [4]). The injection of the $Surv_{90}$ dose in five animals didn't cause any deaths. The toxic effects appeared mainly in the liver and kidneys.

### ACKNOWLEDGEMENTS

The authors are grateful to Fundação de Amparo à Pesquisa do Estado de São Paulo (FAPESP) and to Conselho Nacional de Desenvolvimento Científico e Tecnológico (CNPq) for financial support. One of the authors (BPE) is also grateful to the CNPq for a Ph.D. fellowship grant.

### REFERENCES

[1] see, for example, Souza AR, Najjar R, Glikmanas S, Zyngier SB *J.Inorg. Biochem.* 1996, **64**, 1-5; Pruchnik FP, Bien M, Lachowicz T *Met.-Based Drugs* 1996, **3**, 185-195; Pruchnik FP, Kluczewska G, Wilczok A, Mazurek U, Wilczok T *J. Inorg. Biochem.* 1997, **65**, 25-34; Bien M, Lachowicz TM, Rybka A, Pruchnik FP, Trynda L *Met.-Based Drugs*, 1997, **4**, 81-88

[2] Souza, AR; Najjar, R; Oliveira, E; Zyngier, SB *Met.-Based Drugs* 1997, **4**, 39-41

[3] Craciunescu, DG; Molina, C; Parrondo, E; Alonso, MP; Lorenzo, C; Doadrio, JC; Gutierrez, MT; Frutos, MI; Gaston, E; Certad, G; Ercoli, N *An. Real Acad. Farm.* 1991, **57**, 15-36

[4] Espósito, BP; Zyngier, SB; Souza, AR; Najjar, R *Met.-Based Drugs* 1997, **4**, 333-338

[5] Skippes, HE; Schmidt, LH *Cancer Chemother. Rep.* 1962, **17**, 1-143

**Received: September 28, 1998 - Accepted: October 9, 1998 - Received in revised camera-ready format: December 18, 1998**

